# Pneumatospinning Biomimetic Scaffolds for Meniscus Tissue Engineering

**DOI:** 10.3389/fbioe.2022.810705

**Published:** 2022-02-02

**Authors:** Erik W. Dorthé, Austin B. Williams, Shawn P. Grogan, Darryl D. D’Lima

**Affiliations:** ^1^ Department of Orthopaedics, Shiley Center for Orthopaedic Research and Education, Scripps Health, San Diego, CA, United States; ^2^ Intitute for Biomedical Sciences, San Diego, CA, United States

**Keywords:** pneumatospinning, solution blow spinning, electrospinning, fibrous scaffolds, infra patella fat pad, meniscal tears, meniscal repair

## Abstract

Nanofibrous scaffolds fabricated via electrospinning have been proposed for meniscus tissue regeneration. However, the electrospinning process is slow, and can only generate scaffolds of limited thickness with densely packed fibers, which limits cell distribution within the scaffold. In this study, we explored whether pneumatospinning could produce thicker collagen type I fibrous scaffolds with higher porosity, that can support cell infiltration and neo-fibrocartilage tissue formation for meniscus tissue engineering. We pneumatospun scaffolds with solutions of collagen type I with thicknesses of approximately 1 mm in 2 h. Scanning electron microscopy revealed a mix of fiber sizes with diameters ranging from 1 to 30 µm. The collagen scaffold porosity was approximately 48% with pores ranging from 7.4 to 100.7 µm. The elastic modulus of glutaraldehyde crosslinked collagen scaffolds was approximately 45 MPa, when dry, which reduced after hydration to 0.1 MPa. Mesenchymal stem cells obtained from the infrapatellar fat pad were seeded in the scaffold with high viability (>70%). Scaffolds seeded with adipose-derived stem cells and cultured for 3 weeks exhibited a fibrocartilage meniscus-like phenotype (expressing COL1A1, COL2A1 and COMP). *Ex vivo* implantation in healthy bovine and arthritic human meniscal explants resulted in the development of fibrocartilage-like neotissues that integrated with the host tissue with deposition of glycosaminoglycans and collagens type I and II. Our proof-of-concept study indicates that pneumatospinning is a promising approach to produce thicker biomimetic scaffolds more efficiently that electrospinning, and with a porosity that supports cell growth and neo-tissue formation using a clinically relevant cell source.

## Introduction

Menisci are an essential tissue in knee joints that contribute to load distribution, knee stability, and protect articular cartilage during normal activity. Injuries to menisci are common, with an estimated incidence between 600,000 and 850,000 in the United States alone, 90% of which require surgical intervention (Milachowski et al., 1989; DeHaven, 1999; Turman and Diduch, 2008). Tears occurring in the vascular zone have a higher rate of repair *via* surgery (Makris et al., 2011). Very few tears in the avascular zone heal, and around a third of repairs fail. Smaller tears are frequently repaired using sutures, screws, arrows or darts (Fillingham et al., 2017; Ardizzone et al., 2020). However, many tears are too complex or extensive to repair and thus most surgeries involve partial, subtotal or total meniscectomy (Ford et al., 2005; Fetzer et al., 2009). Loss of meniscal tissue alters joint loading dynamics, leading to joint destabilization and finally a progression to osteoarthritis (OA) (Englund et al., 2001; Lohmander et al., 2007; Rai et al., 2020; Rodeo et al., 2020). To address large tears or for the replacement of menisci, meniscal substitutions have been proposed for partial and total meniscus replacement (Winkler et al., 2020).

Efforts towards engineering meniscus tissues typically combine cells (Angele et al., 2021) with a variety of diverse natural and synthetic scaffolds (Kwon et al., 2019; Li et al., 2021). However, a central feature that underlies the load bearing role of meniscus is the unique organization of collagen fibers, which is an essential requirement for a functional engineered meniscus. To this end, we, and other groups, have applied electrospinning (ES) to create nanofibrous scaffolds that emulate the meniscus collagen fibrillar matrix using natural and synthetic polymer (Grogan et al., 2020; Wang et al., 2021).

Examples of ES scaffolds explored for meniscus regeneration include synthetic polymers such as polylactic acid (PLA) (Baek et al., 2015), polycaprolactone (PCL) (Baker and Mauck, 2007; Ionescu et al., 2012; Gopinathan et al., 2015), natural polymers such as collagen (Baek et al., 2016, Baek et al., 2018), or combinations such as PCL and silk fibroin (Li et al., 2020), poly(lactic-co-glycolic) acid (PLGA) and gelatin (Li et al., 2016), or PLA and collagen (Baek et al., 2019). These electrospun scaffolds were biocompatible, supported attachment of a variety of cell types including meniscus fibroblasts (Kang et al., 2006; Martinek et al., 2006; Puetzer et al., 2015; Baek et al., 2020), various sources of MSC (Pak et al., 2014; Oda et al., 2015; Chew et al., 2017; Baek et al., 2018; Sasaki et al., 2018; Rothrauff et al., 2019) and synovial cells (Horie et al., 2009; Kondo et al., 2019; Sekiya et al., 2019), and lead to neo-tissue formation and repair of *ex vivo* meniscal tears (Baek et al., 2016; Baek et al., 2019). Electrospun scaffolds show promise in terms of cytocompatibility and neotissue formation, however, electrospinning often requires days to generate a scaffold of even a few millimeters in thickness (Dos Santos et al., 2020). Layering of ES scaffolds (Baek et al., 2015; Fisher et al., 2015) or incorporating ES collagen micro/nanofibers within a macroporous PLA/PLGA foam have been described (Bahcecioglu et al., 2014) to generate thicker constructs. However, the issue of delamination under loading remains a concern. Moreover, the highly dense nature of the fibers comprising the scaffold hinder efficient cell seeding and migration throughout the scaffold. To overcome this issue, strategies have been adopted to increase scaffold porosity by including water soluble sacrificial fibers (Baker et al., 2012; Shimomura et al., 2015), or by incorporating growth factors or chemoattractants within the scaffold to facilitate cell migration, support proliferation and tissue formation (Qu et al., 2017; Qu et al., 2019; Baek et al., 2019).

Solution blow spinning or pneumatospinning is an alternate method of generating fibrous scaffolds. Pressurized gas driven through an outer nozzle generates a stream of polymer solution fed through the inner nozzle of a coaxial system (Dos Santos et al., 2020). Pneumatospinning relies on gas pressure instead of an electric charge, which facilitates the fabrication of thicker constructs without the insulating effect that limits the thickness of electrospun constructs. Pneumatospinning is reportedly simpler, safer, more efficient, and can be achieved with less expensive and commercially available tools and therefore has the potential to overcome some of the limitations of electrospinning for meniscal repair (Behrens et al., 2014; Daristotle et al., 2016; Polk et al., 2018; Molde et al., 2020).

A wide range of materials have been pneumatospun to create nano- and microfibrous scaffolds. While pneumatospinning of synthetic polymers such as polyurethane (PU), polymethylmethacrylate, polyvinyl alcohol, PLA, and PCL is most commonly reported (Medeiros et al., 2009; Oliveira et al., 2011; Srinivasan et al., 2011; Behrens et al., 2014; Santos et al., 2016; Tomecka et al., 2017; Akentjew et al., 2019; Kopec et al., 2020); natural polymers such as silk fibroin and collagen have also been successfully pneumatospun (Magaz et al., 2018; Polk et al., 2018).

We therefore explored the potential of pneumatospinning for rapid generation of microfibrous collagen scaffolds seeded with human infrapatellar fat pad progenitors (IPFP-MSC) to facilitate meniscogenesis. We characterized scaffold microstructure, mechanical properties, and cytocompatibility. We analyzed cell migration and distribution, neotissue formation, and assessed proof of concept in the repair of meniscal tears in *ex vivo* bovine and human osteoarthritic menisci.

## Materials and Methods

### Pneumatospinning System

**FIGURE 1:**
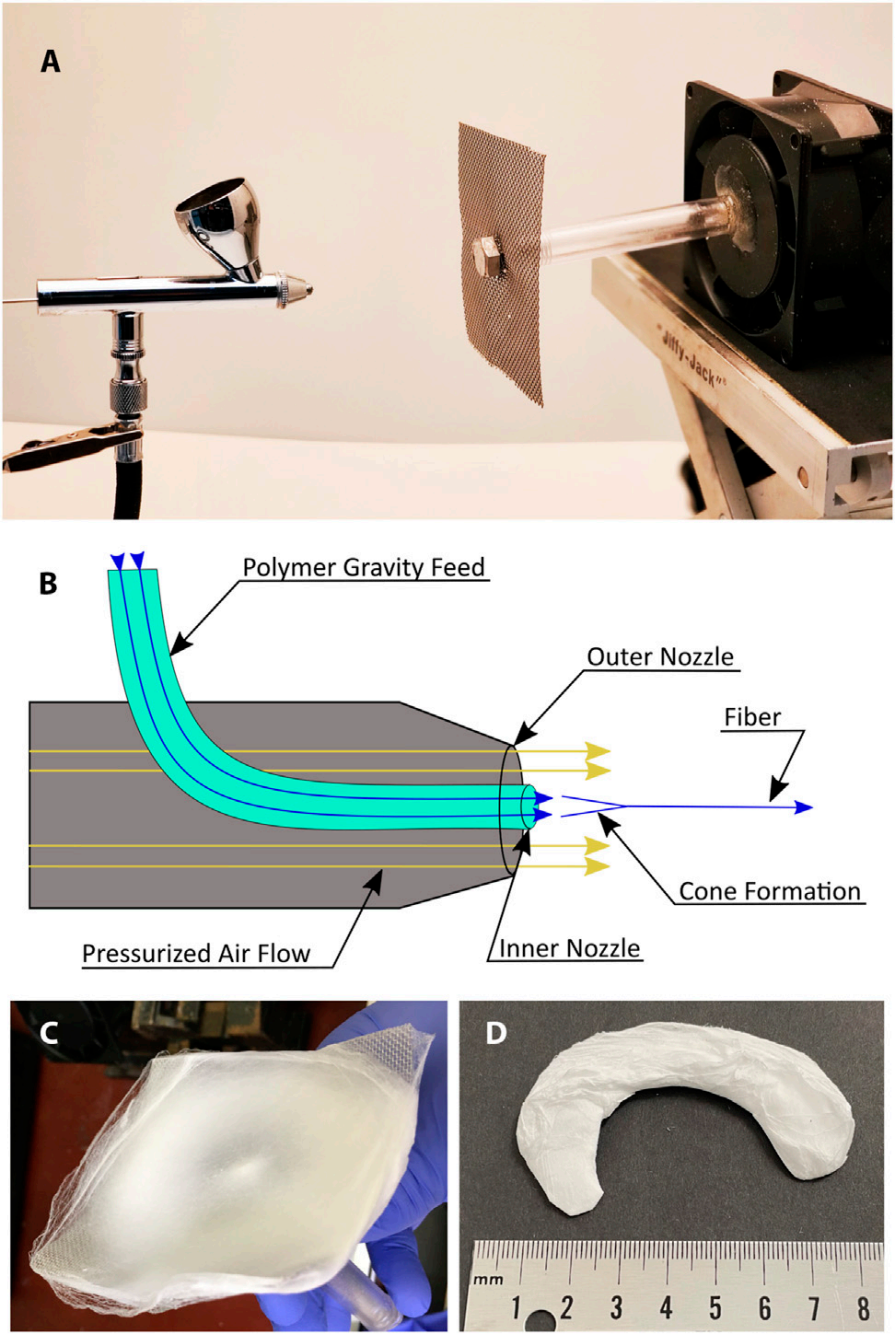
Pneumatospinning setup **(A)** Photograph of airbrush and mesh collector. **(B)** Diagram of polymer jet generated by air flow. **(C)** Photograph of scaffold spun on mesh collector. **(D)** Scaffold cut to the shape of a human meniscus.

Collagen type I (Semed S, generously supplied by DSM Biomedical, Exton, PA) was dissolved in hexafluoroisopropanol (HFIP) at concentrations between 5 and 10% wt/vol to establish the optimal concentration for spinning. The collagen solution was loaded into a customized gravity feed airbrush with a 350 µm nozzle (Anest Iwata, Yokohama) modified to run continuously. The airbrush consisted of an inner feed line, fed by gravity, leading to a nozzle and an outer air line, ending in a cap to form a coaxial outlet with the nozzle (Figure 1). The low pressure created by the escaping air drew solution from the inner nozzle. Air pressure on the air brush was set to 30 psi.

The target was a square section of flat stainless-steel mesh with a .03 cm wire diameter (TWP Inc., Berkeley) mounted on an electric motor and rotated at 650 RPM with the axis of rotation co-linear with the axis of the airbrush nozzle. The distance between the nozzle and target was set to 12 cm (Figure 1). The shape of the target mesh was varied by introducing 3-dimensional curvatures to assess potential for spinning a net meniscal shape (Supplementary Figure S1).

For comparison with electrospinning, scaffolds were fabricated as previously reported (Baek et al., 2016). Briefly, a collagen solution of 20% wt/vol was extruded toward a rotating drum with a potential difference of 18 kV.

### Glutaraldehyde Crosslinking of Collagen Scaffolds

Collagen scaffolds were subjected to glutaraldehyde (GA, 25%, Thermo Fisher Scientific, Waltham, MA) vapor to induce crosslinking. We had previously used glutaraldehyde (GA) for electrospun collagen and found this to be an effective means of crosslinking collagen scaffolds (Baek et al., 2016; Baek et al., 2018). Collagen scaffolds on the collector mesh were suspended over an open beaker containing 30 ml GA solution and enclosed in a glass container for 48 h to contain the GA fumes in a chemical fume cabinet at 40°C. After GA crosslinking, scaffolds were cut to size and washed in PBS four to five times and stored in PBS at 4°C.

### Mechanical Properties

**FIGURE 2:**
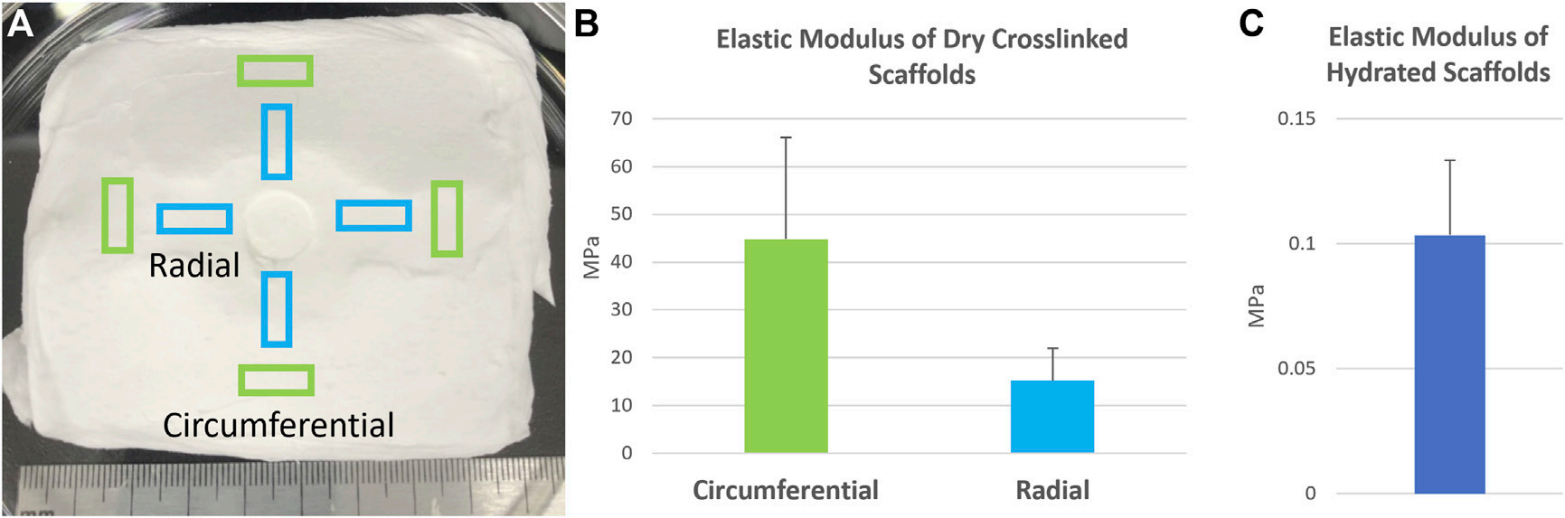
Mechanical properties of pneumatospun collagen scaffolds deposited upon a spinning mesh collector. **(A)** Scaffold specimens were harvested from the mesh collector in either in the circumferential or radial directions in relation to the rotation of the scaffold. **(B)** Elastic modulus of dry collagen scaffolds after glutaraldehyde crosslinking and **(C)** following hydration in medium. Error bars represent standard deviations.

Specimens were tested for tensile strength and tensile modulus using an Instron 8511 servohydraulic testing machine (Instron, Norwood, MA). Briefly, rectangular sections of 8 mm by 26 mm (*n* = 5) were cut from dry pneumatospun mats with the long axis tangent to the rotation of the collector (circumferentially oriented) or the long axis radial to the rotation of the collector (radially oriented, Figure 2). To test the effect of hydration, specimens were hydrated in 1x PBS for 1 h before mechanical testing (*n* = 6). The thickness and width of each specimen was measured with a digital caliper immediately prior to mechanical testing. Gauge length was measured for each specimen after mounting in the tensile grips. Specimens were mounted with a set of Instron grips and loaded to failure under tension at 4.2 mm/s. Load and displacement were recorded throughout the loading process.

### Scanning Electron Microscopy (SEM)

For SEM, scaffolds were coated with 3.7 nm of Iridium and imaged on a Hitachi S-4800 SEM at 3 kV and 8 mm WD. SEM images were assessed for porosity and fiber morphology using ImageJ software (NIH, Bethesda, MD). Porosity was measured using ASTM F1854-15 (ASTM, 2015). Briefly, images were segmented to separate foreground fibers from background. Four lines were drawn horizontally across the image and values were measured along the corresponding profile. The percentage of pore space along these profiles was averaged to arrive at a value for the image. Pore size was measured across the longest axis of the pores to calculate average pore size in the transverse plane.

### Cell Harvesting and Culture

Infrapatellar fat pad (IPFP) tissue was obtained from patients (74 year old female and 75 year old male) undergoing total knee replacement (approved by Scripps Institutional Review Board). Mesenchymal stem cells (IPFP-MSC) were isolated using a previously described method (Grogan et al., 2020). Briefly, IPFP tissues were minced using a scalpel to create fragments (∼5 mm^3^), which were placed into 6-well plate wells precoated with human collagen type I (Cell Adhere, StemCell Technologies, Vancouver, Canada). For the first 12 h, the tissue fragments were maintained in a CO_2_ incubator at 37°C in only .5 ml MSC-medium (LONZA, Walkersville, MD) supplemented with Fibroblast Growth Factor 2 (FGF-2) (10 ng/ml; PeproTech, RockyHill, NJ). After 12 h, 1.5 ml of medium was added and the tissue fragments were cultured for 1–2 weeks until emergence of cells from the tissue. The remaining tissue fragments were discarded, and the emerging cells were detached using Accutase (Innovative Cell Technologies, Inc. San Diego, CA) and reseeded into collagen coated flasks at a density of 350,000 cells per cm^2^.

### Scaffold Cell Seeding and Culture Conditions

Pneumatospun and GA cross-linked collagen scaffolds were cut into rectangular specimens (approx. 1 cm by .5 cm) and washed in PBS extensively (at least 5 times in 5 ml PBS over 24 h). These scaffolds were seeded with IPFP-MSC (.5 × 10^6^ cells per scaffold specimen), cultured in MSC medium, and placed upon an orbital shaker at 100 RPM (Ohaus Parsippany, NJ) in a CO_2_ incubator at 37°C. The seeded scaffolds were maintained in MSC medium for 3–4 days for initial cell proliferation before changing to differentiation medium that consisted of Dulbecco’s Modified Eagle Medium (DMEM) (Mediatech Inc., Manassas, VA), 1x ITS+1 supplement (Sigma-Aldrich, St. Louis, MO), 100 nM Dexamethasone (Sigma), 1.25 mg/ml human serum albumin (Bayer, Leverkusen, Germany), 100 µM ascorbic acid 2-phospahate (Sigma), 1% penicillin/streptomycin/gentamycin (PSG, Gibco, Carlsbad, CA) and supplemented with 10 ng/ml TGFβ3 (PeproTech, Rocky Hill, NJ). Scaffolds were cultured for 3 weeks (with medium changes every 3–4 days) to assess cell attachment, viability, and gene expression.

### Cell Viability Assessments

A total of 11 seeded scaffolds were used to assess viability using two different donors after 21 days in culture. Cell viability and distribution after 24 h or following 3 weeks in culture was measured with the Live/Dead kit (Invitrogen, Waltham, MA). The staining buffer consisted of Ethidium Homodimer-1 (8 mM) and Calcein-AM (1.6 mM) suspended in PBS. The cell seeded collagen scaffolds were incubated for 30–40 min before visualization with either a fluorescence microscope (Axiovert 200M, Zeiss, Jena, Germany) or via a confocal laser microscope (LSM-810; Zeiss). Image processing by thresholding and segmentation was used to remove the background scaffold stain (Supplementary Figure S2). The percentage of live and dead cells was calculated using ImageJ/Fiji (Rueden et al., 2017).

### *Ex vivo* Meniscus Repair Models

*Bovine explants:* Whole bovine knees with the knee capsule intact were obtained from Animal Technologies, Inc. (Tyler, TX). Meniscal tissue explants of approximately 1 cm wide and x 3 cm deep were cut under sterile conditions. Explants were cultured in DMEM with 10% CS and 1% PSG until ready to use.

*Human explants:* Menisci were obtained from four patients (69.3 ± 10.1 years, three female, one male) following total knee arthroplasty (approved by Scripps Institutional Review Board). The menisci were cut into tissue explants of around 1 cm × 1 cm and each cultured in DMEM with 10% CS and 1% PSG until ready for scaffold implantation (within 2 days).

Collagen scaffolds (approx. 1 cm long, .5 cm wide, and .2 cm thick) were seeded with IPFP-MSC (.5 × 10^6^ per scaffold) and cultured for 3 days in MSC-medium, followed by 7 days in differentiation medium. Scaffolds (*n* = 29) were implanted to repair surgically created longitudinal or transverse defects in human (*n* = 4 human donors, *n* = 17 explants) and bovine explants (*n* = 2 knees, *n* = 12 explants). The longitudinal defects were created in the red-white region and the transverse defects spanned the red-white regions. The implanted scaffolds and explant tissue was cultured for 3 weeks in the differentiation medium, with changes every 3–4 days. At the end of culture, the explants were fixed and processed for paraffin embedding and subsequent histological analyses.

### Histology and Immunohistochemistry

Paraffin embedded sections (4 μm thick) were mounted on glass slides for staining with Safranin O-fast green or immunohistochemistry for collagen types I and II as previously described (van Schaik et al., 2021). Briefly, paraffin cut sections were deparaffinized and treated with pepsin for 9 min at 37°C in a humidified chamber (Digest-All 3, Thermo Fisher Scientific). Rabbit anti-human collagen type I antibody (1 μg/ml; Ab 34,710, Abcam, Cambridge, MA) and mouse anti-human collagen type II (2 μg/ml; II-II6B3, Hybridoma Bank, University of Iowa) was used as the primary antibodies, which were incubated at 4°C for 12–16 h in a humid chamber. The ImmPRESS secondary DAB (Brown) or AP (red) kits were used for color development (Vector Laboratories, Burlingame, CA). Non-specific staining was evaluated using species-matched isotype controls at the same concentration as the specific primary antibodies.

### Gene Expression

Following the manufacturer’s instructions (Qiagen, Valencia, CA), scaffolds were minced by scalpel in RLT buffer and then subjected to homogenization using the Qiashredder. Total RNA was extracted using the RNeasy kit (Qiagen) and cDNA was made using the High Capacity cDNA Reverse Transcription Kit (Applied Biosystems, Foster City, California). Verified primer/probe assays were purchased from Applied Biosystems to monitor the expression levels of COL1A1, COL2A1, COMP, ACAN, and THY1 relative to the GAPDH (housekeeping gene). The gene expression levels of IPFP-MSC in monolayer culture were used to calculate relative changes in expression level following culture on the pneumatospun collagen scaffolds. Gene expression was normalized to GAPDH and we used the ΔCt method as previously reported (Martin et al., 2001).

### Statistical Analysis

Difference in gene expression between IPFP-MSC cultured on pneumatospun scaffolds and cells in monolayer cultures were tested for significance at *p* < .05, using the online BootstRatio application (http://regstattools.net/br) (Cleries et al., 2012).

## Results

### Efficient Fabrication of Microfibrous Collagen Scaffolds

Pneumatospinning involves a gravity-fed airbrush which generated undesirable turbulence at traditional collector targets used in electrospinning such as flat plates or solid drums. We therefore selected meshed collectors that permit air flow through the target, reduce turbulence, and result in more consistent accumulation of spun fibers. To further reduce turbulence at the target, we mounted a fan behind a stationary collector to direct air through the target and mounted the target to the spinning fan to induce circumferential alignment of fibers.

We tested a range of collagen solution concentrations between 5 and 10% wt/vol to identify the optimal solution that could be reliably dispensed. Solutions below 6% formed insufficient fibers, while those above 9% tended to clog the nozzle of the airbrush. A 9% solution was chosen for all following experiments. 60 ml of collagen solution generated a 1 mm thick scaffold of collagen fibers in less than 2 h. This was significantly faster than our electrospinning experiments which required over 24 h to generate 250 µm thick scaffolds.

We also demonstrated the potential to create scaffolds of varying shapes, using curved targets with radial ripples (Supplementary Figure S1). Glutaraldehyde crosslinked mats spun onto these targets retained the curvature of the targets after removal, but showed no appreciable variation in thickness. For subsequent experiments in this study, we used flat square mesh collectors.

### Scaffold Properties

Before crosslinking, spun collagen mats were fragile, fragmented upon removed from the target substrate, and dissolved in culture. After glutaraldehyde crosslinking, collagen mats could be easily removed intact from the target. By 3 weeks, cultured scaffolds swelled and softened, but retained their net spun shape and could be easily manipulated with forceps for loading into mechanical testing fixtures or for surgical implantation in *ex vivo* tissue samples.

**FIGURE 3:**
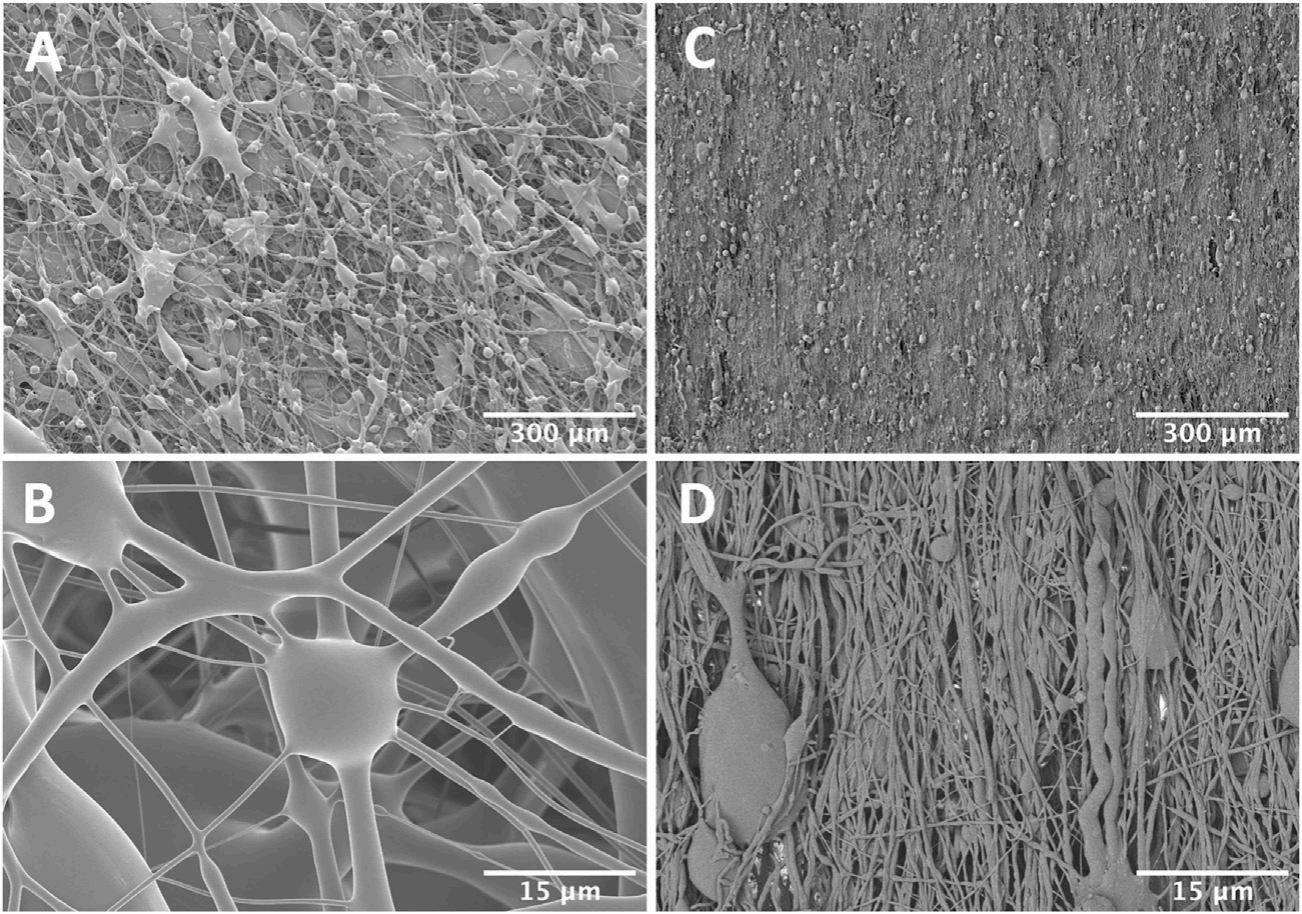
Scanning electron micrographs of pneumatospun **(A,B)** or electrospun **(C,D)** collagen scaffolds after glutaraldehyde crosslinking. Note the larger interconnected porosities in the pneumatospun scaffolds.

Dry collagen GA crosslinked scaffolds exhibited an elastic modulus of around 45 MPa, which was reduced significantly after hydration to .1 ± .03 MPa (Figure 2). Dry scaffolds failed consistently within the gauge length of the test setup, while hydrated scaffolds tended to fail near the tissue grips. SEM imaging of the GA crosslinked pneumatospun scaffolds revealed a heterogenous distribution of fiber sizes and large, interconnected porosities (Figure 3). This contrasted with aligned, electrospun scaffolds which have low porosity and more uniform, nanoscale fibers (Figure 3). Pneumatospun fibers appeared irregular with diameters ranging from 1 to 30 μm. The overall porosity of the collagen scaffold in the transverse plane was approximately 48% with pore sizes ranging from 7.4 to 100.7 µm with a median of 23.8 µm and a mean of 25.7 µm. We also observed bead formation which is related to the surface tension of the solution and can be reduced by optimizing solution and spinning parameters.

### Neotissue Formation

**FIGURE 4:**
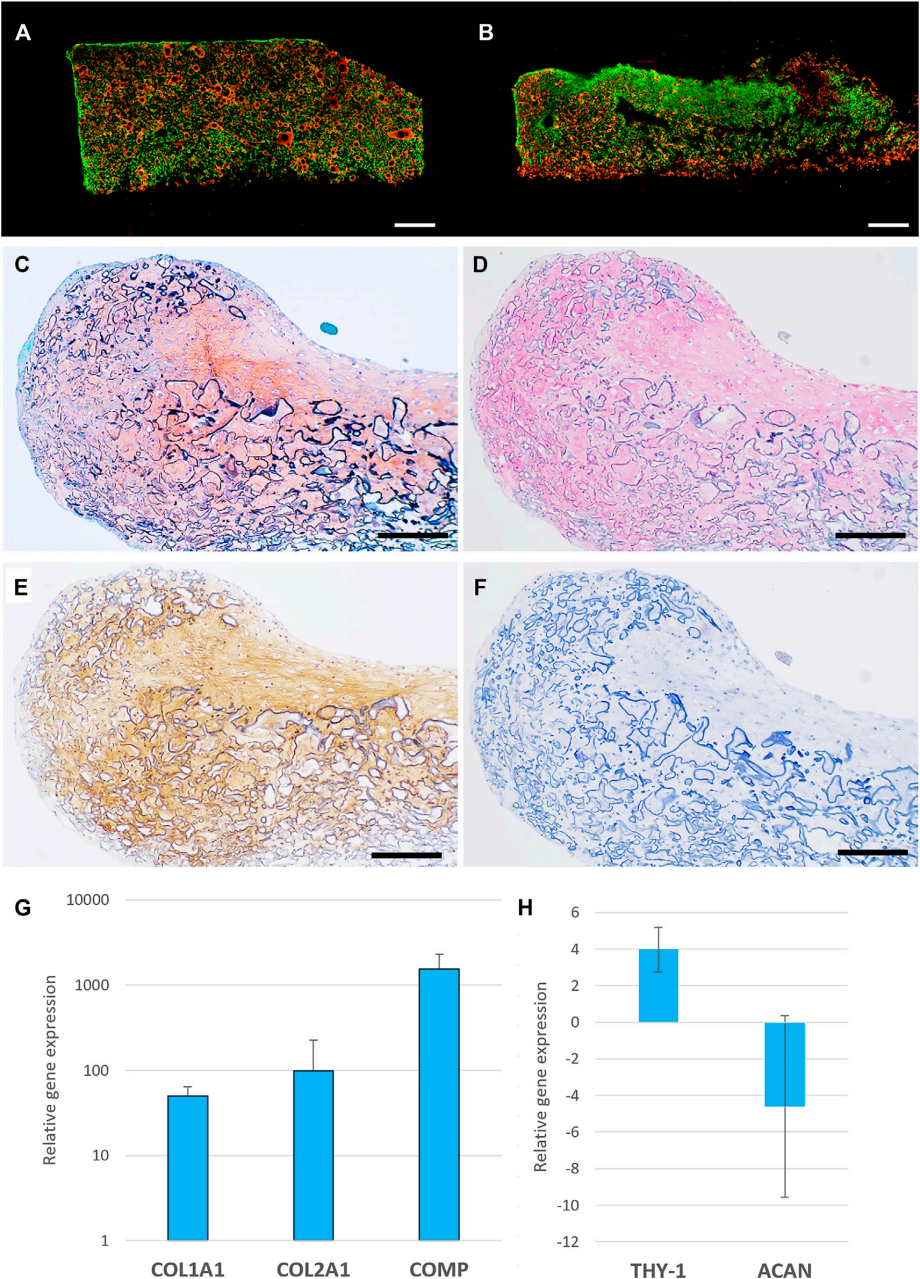
Cell viability, histology and gene expression of IPFP-MSC seeded pneumatospun collagen scaffolds. **(A,B)** Cell viability of IPFP-MSC seeded collagen scaffolds (live cells = green), dead cells = red, bar = 1000 μm) **(C)** Safranin O stain of neotissue formed on collagen scaffold. **(D)** Collagen type I immunostaining (AP red). **(E)** Collagen type II immunostaining (DAB brown). **(F)** Isotype control immunostaining. (Bar = 200 μm) **(G,H)**. Gene expression levels of IPFP-MSC seeded on collagen scaffolds after 3 weeks in serum free medium relative to monolayer undifferentiated cultured IPFP-MSC (*n* = 6).

Glutaraldehyde crosslinked pneumatospun collagen scaffolds were seeded with IPFP-MSC, cultured in 6-well plates in MSC medium and placed on an orbital shaker overnight in the incubator. This approach enhanced cell attachment and infiltration into the scaffold (Supplementary Figure S3). After 3 weeks of culture in serum-free differentiation medium, IPFP-MSC were observed throughout the pneumatospun collagen scaffolds with 70.2 ± 7.5% viability (Figures 4A,B).

On histological analysis, IPFP-MSC seeded into the scaffolds generated a fibrocartilage-like neotissue with extracellular matrix containing glycosaminoglycans (Safranin O positive; Figure 4C), collagen types I and II (Figures 4D–F). This histology was generally consistent with the gene expression profile of increased COL1A1, COL2A1 and COMP, THY-1 (CD90) and reduced ACAN expression compared to IPFP-MSC in monolayer culture (Figures 4G,H).

### Repair of Ex Vivo Meniscus Tears

**FIGURE 5:**
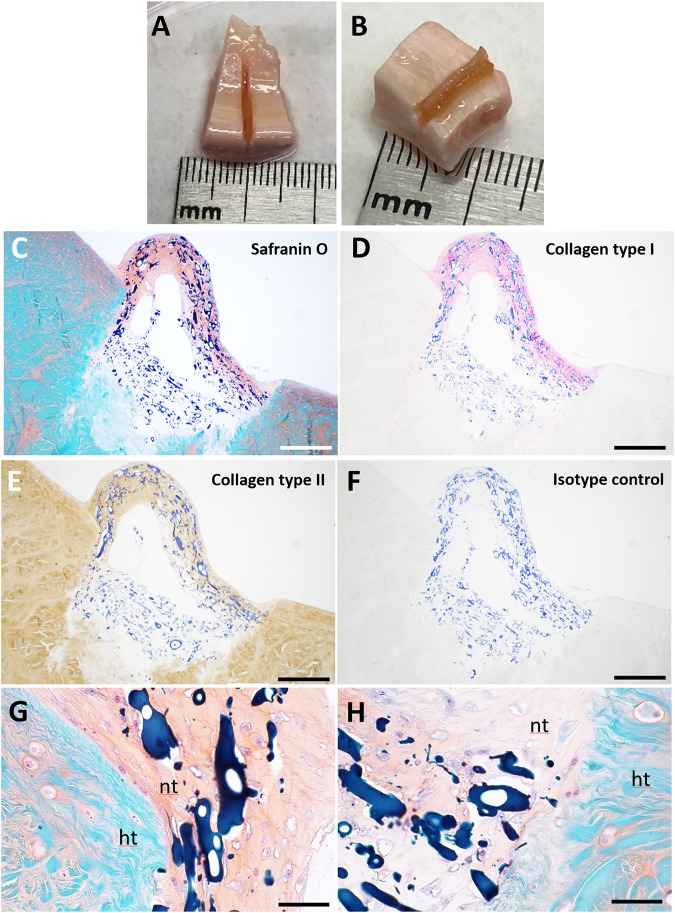
Pneumatospun collagen scaffolds seeded with human IPFP cells and implanted into *ex vivo* bovine meniscus and culture for 3 weeks. **(A,B)** Photographs of implanted collagen scaffolds. **(C)** Safranin O staining. **(D)** Collagen type I immunostaining. **(E)** Collagen type II immunostaining. **(F)** Isotype control. **(G,H)** Higher magnification of Safranin O stained image showing integration of neotissue (nt) with host tissue (ht). The collagen scaffold is stained dark blue. (C-F Bar = 200 μm; G-H Bar = 50 μm).

**FIGURE 6:**
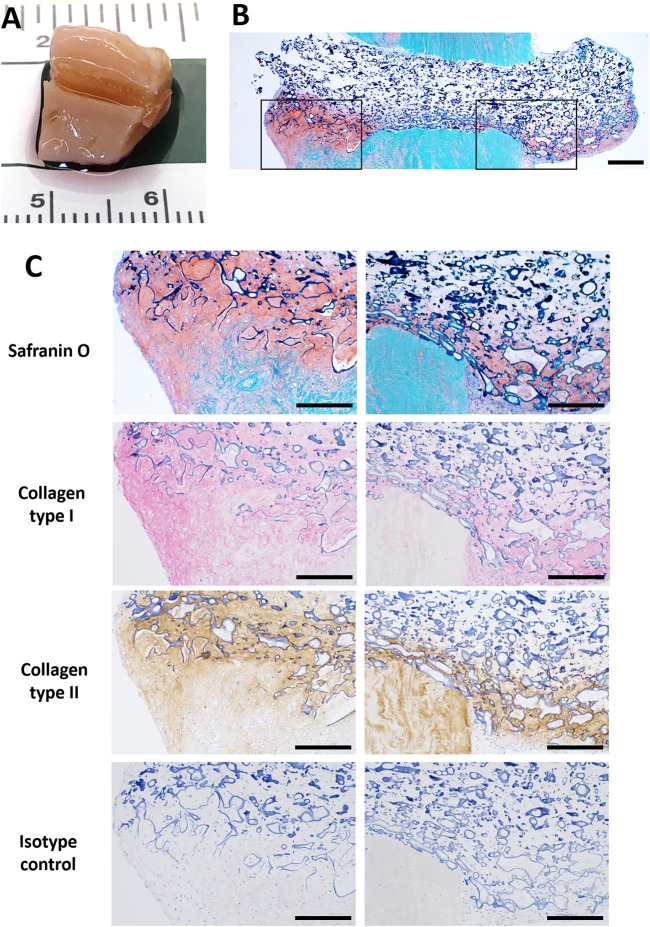
Pneumatospun collagen scaffolds seeded with human IPFP cells and implanted into human OA meniscus explant (72 year-old female) and cultured for 3 weeks. **(A)** Photograph of implanted scaffold. **(B)** Overview of section stained with Safranin O showing neotissue development in meniscus defect (bar = 500 μm). **(C)** Histologic images of sections stained with Safranin O and immunostained with collagen type I (AP red), collagen type II (DAB brown), and isotype control regions of inset regions outlined in **(B)** (bar = 300 μm).

We assessed potential for repair of meniscus tears by implanting scaffolds seeded with IPFP-MSC into surgically created defects in normal healthy bovine meniscus (Figures 5A,B) or in osteoarthritic human meniscus resected from patients that had undergone TKA (Figure 6A). After 3 weeks of culture in differentiation medium, neo-fibrocartilaginous tissue developed in the bovine meniscus that integrated with the host tissue (Figure 5). More GAG deposition was noted in the superficial region corresponding spatially with the GAG seen in the host tissue (Figure 5C). The mix of collagen types I and II indicated a fibrocartilage-like composition. Cell-seeded collagen scaffolds implanted into osteoarthritic human menisci (Figure 6A) also resulted in similar fibrocartilage-like neotissue which integrated with host tissue with a positive Safranin O and a collagen type I and II staining profile (Figures 6B,C).

## Discussion

We explored pneumatospinning to fabricate microfibrous collagen scaffolds as a more efficient approach to electrospinning (ES) for meniscal tissue engineering, with potential for greater porosity to facilitate cell seeding and migration, generation of meniscus-like tissue, and to repair meniscal tears. We demonstrated that pneumatospinning can rapidly produce scaffolds with a spectrum of micron-sized fibers and appropriate porosity. Pneumatospun collagen scaffolds were biocompatible, and permitted rapid IPFP-MSC attachment and colonization, and formation of neo-fibrocartilage meniscus-like tissue, especially when implanted into healthy bovine or osteoarthritic human meniscus explants.

Several synthetic polymers (polymethylmethacrylate, polylactic acid, and polystyrene) have been pneumatospun to produce micro- and nano-fibers up to 100x faster than electrospinning) (Medeiros et al., 2009). The authors found that electrospinning typically dispensed solutions between 4 and 10 μl/min while pneumatospinning dispensing rates can be increased up to 200 μl/min without altering fiber diameter (Medeiros et al., 2009). Most other studies did not report on the final thickness of the deposited scaffold or the accumulation rate over a given time. However, pneumatospun fiber mats are reported to take a few seconds to minutes to build a mat of significant thickness with deposition rates of .1 ml/min or more (Medeiros et al., 2009; Oliveira et al., 2011; Behrens et al., 2016). Others have reported pneumatospun mat thickness ranging from 130 µm to 1 mm (Behrens et al., 2016; Kopec et al., 2020; Molde et al., 2020). Polk et al. (2018) rapidly pneumatospun 200 μm thick collagen type I scaffolds in 5 min, which was 32x faster than electrospun scaffolds. To our knowledge, this is the first study reporting on pneumatospinning for fabricating scaffolds for meniscus regeneration. We generated scaffolds at approximately .5 mm in thickness/hr, which was significantly more efficient than our previous reports on electrospinning which required over 24 h to produce a .25 mm thick electrospun scaffold (Baek et al., 2016); equivalent to a 50x increase in fiber deposition rate.

Pneumatospinning can generate fibers with diameters ranging from 70 nm to as large as 100 μm (Sinha-Ray et al., 2015; Daristotle et al., 2016; Dos Santos et al., 2020) by tuning process variables such as the polymer injection rate and concentration, temperature, air/gas pressure and nozzle geometry (Sinha-Ray et al., 2015; Medeiros et al., 2016; Dos Santos et al., 2020). We pneumatospun collagen fibers ranging from 1 to 30 μm, which were larger than our previous studies with ES collagen fiber (250–860 nm) (Baek et al., 2016). Our pneumatospun collagen fiber diameters were also larger than the average fiber diameter of 224 nm reported for pneumatospun type I atelocollagen scaffolds (Polk et al., 2018). This difference in fiber diameters could be attributed to differences in collagen (atelocollagen vs. soluble collagen), collagen concentration (40 vs. 9%), solvent (acetic acid vs. HFIP), air pressure (60 psi vs. 30 psi), and collector properties.

Electrospinning typically generates densely packed nanofibrous mats with low porosity which can inhibit cell migration into the scaffolds (Baker et al., 2008). Modifications such as sacrificial fibers or microparticles of polyethylene oxide are required to increase porosity and enhance cell infiltration (Baker et al., 2008; Wang et al., 2013). One advantage of our pneumatospun scaffolds was the inherent increased porosity which enhances potential for cell migration. We noted a porosity of nearly 50% in our scaffolds (with pore sizes averaging 26 µm) which supported cell migration. Others have reported varying pore sizes with pneumatospun materials, ranging from 3 to 6 μm for PU (Kopec et al., 2020); 10–50 μm with E1001 (1 k), a tyrosine-derived polycarbonate (Molde et al., 2020); and 25 μm to over 100 μm for scaffolds of PLA and dimethyl carbonate (Medeiros et al., 2016).

Fiber alignment replicating the microstructure of meniscal tissue is important to engineer tissue with biomimetic properties. We used a rotating mesh collector to direct circumferential fiber alignment. Although, SEM did not reveal differences in alignment of fibers, crosslinked collagen scaffold samples in the circumferential direction were three times stiffer in comparison to samples in the radial direction (15 KPa) from the same scaffold. Increasing the speed of collector rotation or modifying collector shape may enhance fiber alignment (Baek et al., 2015; Baek et al., 2016; Baek et al., 2018). For example, a funnel between the nozzle and the collector has been effective in channeling airflow and aligning in-flight fibers (Magaz et al., 2018); and a cylindrical tube with internal spokes collected aligned pneumatospun fibers (Polk et al., 2018).

The meniscus is composed of a fibrocartilaginous tissue that displays variations in the distribution of ECM molecules particularly collagens type I and II, and GAGs (Makris et al., 2011; Murphy et al., 2019). In our study, seeding IPFP-MSC upon pneumatospun collagen scaffolds increased expression of COL1A1, COL2A1, COMP and THY-1 (CD90). COMP plays a critical role in mediating various ECM component interactions such as fibronectin, many collagens (types I, II IX, XII, and XIV) and proteoglycans such as aggrecan, chondroitin sulfates, and heparan sulfate (Chen et al., 2007; Acharya et al., 2014). The increased gene expression of THY-1 also corresponds with a fibrocartilaginous phenotype (Grogan et al., 2017; Grogan et al., 2018). These increases in meniscogenic gene expression were reflected in development of fibrocartilaginous-like neotissue with distinct collagen type I and II protein deposition in a matrix containing GAGs.

*Ex vivo* meniscus tissue cultures are increasingly being utilized as models to assess *in vitro* repair and regeneration (Cucchiarini et al., 2009; Cucchiarini et al., 2015; Marrella et al., 2018; Resmi et al., 2020). We previously characterized an *ex vivo* meniscus explant model of longitudinal tears repaired with collagen ES scaffolds seeded with human meniscus cells (Baek et al., 2016; Baek et al., 2019) and documented composition of neotissue and integration with host explant. In the present study, implantation of thicker pneumatospun scaffolds seeded with IPFP-MSC into meniscal defects resulted in fibrocartilaginous tissues composed of GAGs and collagen types I and II. The scaffolds used in this study may therefore be more suitable for supporting the development of avascular meniscus-like tissues, that typically contain higher concentrations of collagen type II and GAGs than the vascular meniscus regions.

Despite the promising results with pneumatospun collagen scaffolds, there are limitations to overcome. Hydration of glutaraldehyde crosslinked collagen scaffolds in culture significantly decreased mechanical properties, reducing its potential for broader clinical applications such as meniscus replacement. Incorporation of artificial polymers, such as PLA, may increase the mechanical properties as we and others have previously demonstrated with coaxial electrospun fibers (Tong et al., 2015; Singh et al., 2018; Baek et al., 2019; Baek et al., 2020). Our nozzle to collector arrangement did generate encouraging differences in mechanical properties based on sample orientation but did not result in visible fiber alignment; and alternate approaches need to be explored. Our *in vitro* and *ex vivo* systems provided proof of concept of cytocompatibility, cell distributions neo-tissue formation. However, *in vivo* experiments are needed to validate suitability of translation of tissue engineered scaffolds for meniscus repair.

## Conclusion

Pneumatospinning is a promising approach to rapidly generate collagen fibers for meniscus tissue engineering with significantly greater efficiency of scaffold fabrication than electrospinning. The increased porosity effectively supported IPFP-derived cell colonization and differentiation. The resultant neo-fibrocartilage tissue integrated into normal bovine and arthritic human menisci defects. For translation to clinical applications, enhanced mechanical properties and validation in clinically relevant meniscal repair and regeneration models are required.

## Data Availability

The original contributions presented in the study are included in the article/Supplementary Material, further inquiries can be directed to the corresponding author.

## Supplementary Material

Supplementary Figure S1 Photographs of different collector shapes. **(A)** Flat mesh **(B)** Curved mesh **(C,D)** 3D curved shapes.

Supplementary Figure S2 Cell viability of IPFP-MSC seeded pneumatospun collagen scaffolds after overnight incubation on orbital shaker. **(A)** Live cells (green) and **(B)** dead cells (red) (Magnification 10x).

Supplementary Figure S3 ImageJ was used for image processing to remove background staining and extract green (live/cytoplasm) and dead (red /nuclei) cells for viability assessments. **(A)** Confocal microscopy image; **(B)** Live cells labeled green (cell count = 13,773); **(C)** Dead cells labeled red (cell count = 1201): cell viability = 92% (bar = 1000 µm).

Supplementary Figure S4 Immunohistochemistry for collagen types I and II. Left column: Negative isotype controls; Right column: positive stains. **(A,B)** Type I collagen stain for bovine meniscus; **(C,D)** Type I collagen stain for human meniscus; **(E,F)** Type II collagen stain for human articular cartilage.
