# Supplementary figures and images for: Pneumatospinning Biomimetic Scaffolds for Meniscus Tissue Engineering
Source: Front Bioeng Biotechnol. 2022 Feb 2;10:810705. doi: 10.3389/fbioe.2022.810705 (PMC8847752; doi:10.3389/fbioe.2022.810705)

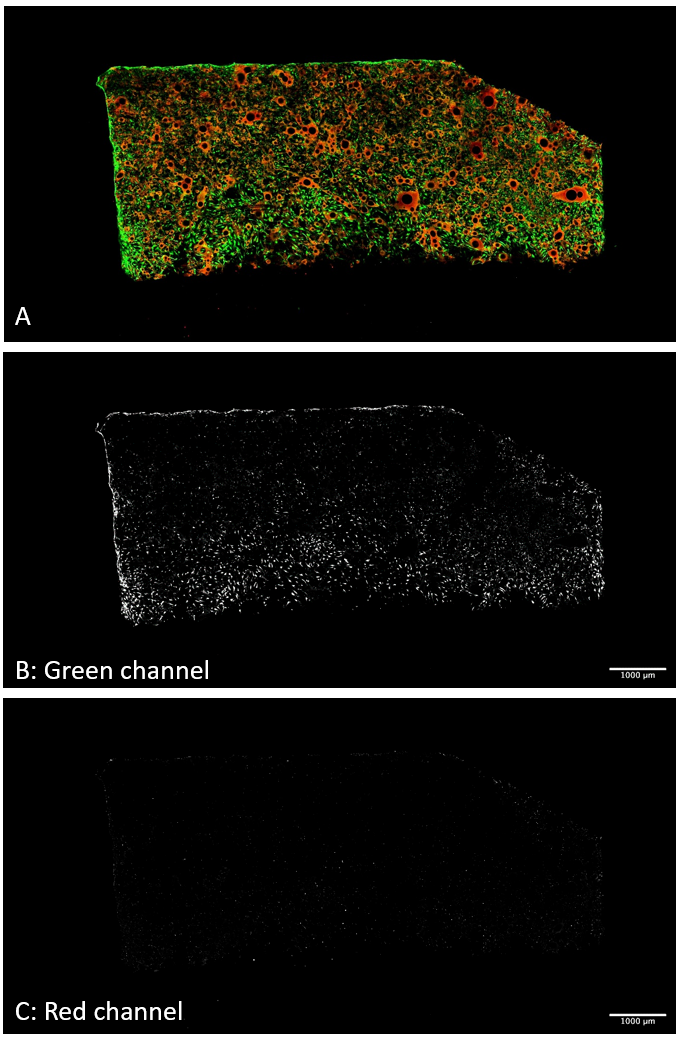

Supplement: Supplementary file 1 [file Image3.TIF]

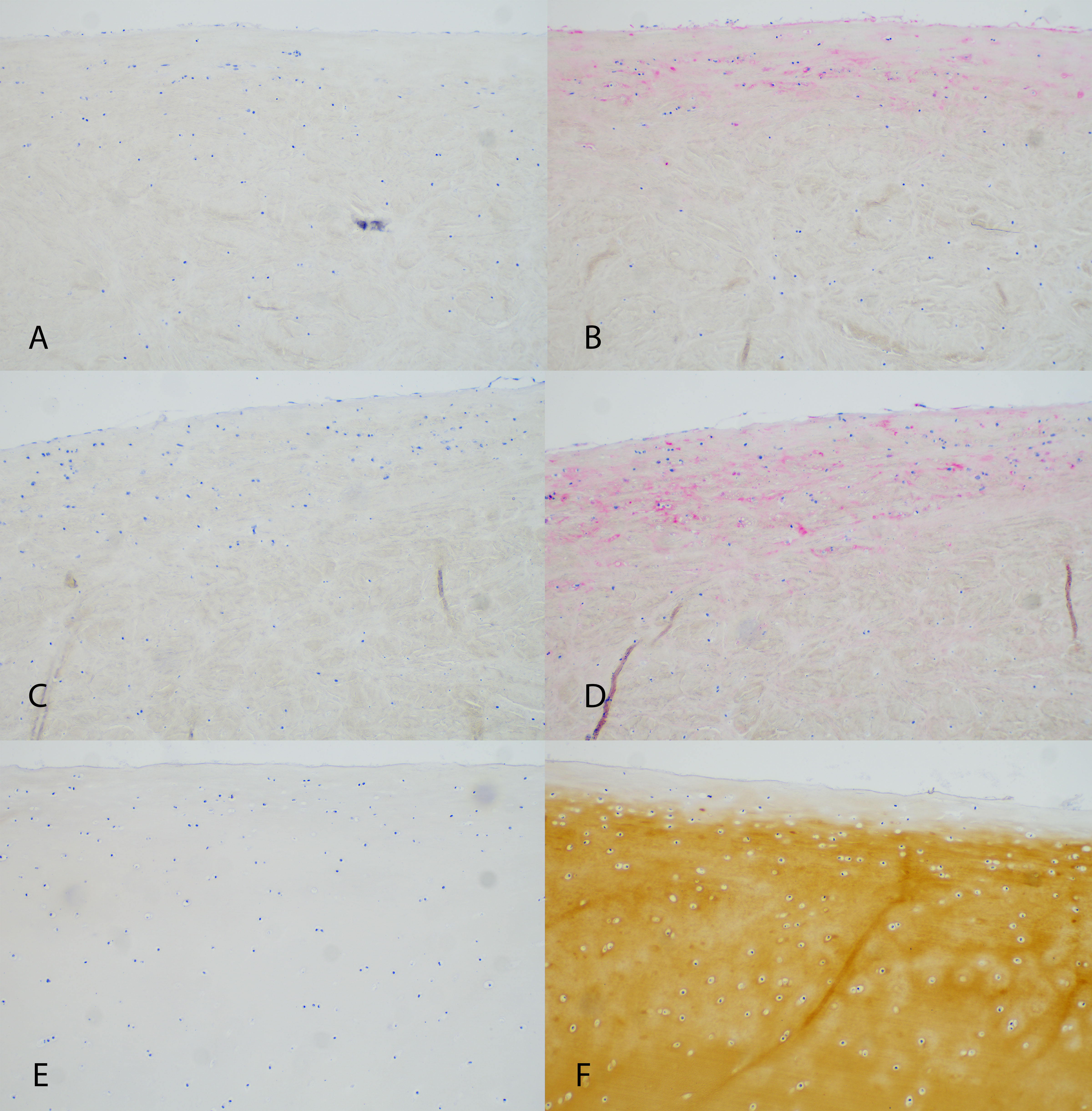

Supplement: Supplementary file 2 [file Image4.JPEG]

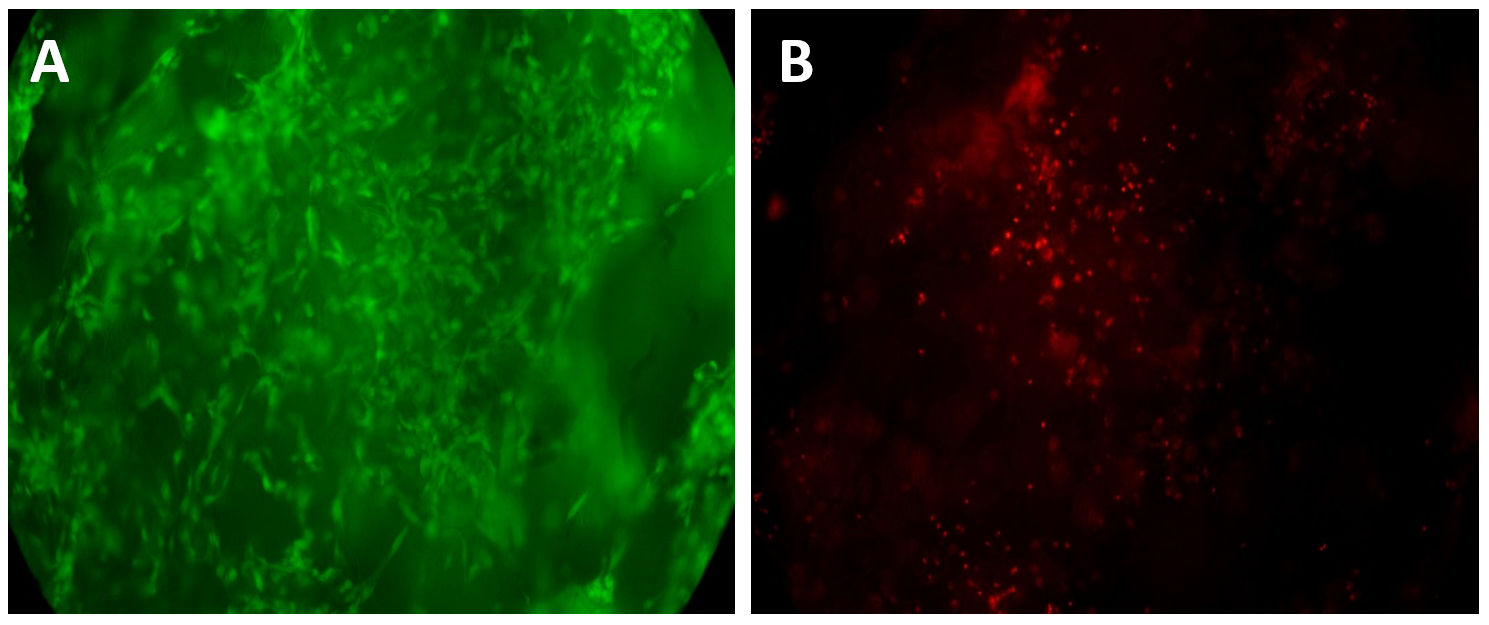

Supplement: Supplementary file 3 [file Image2.TIF]

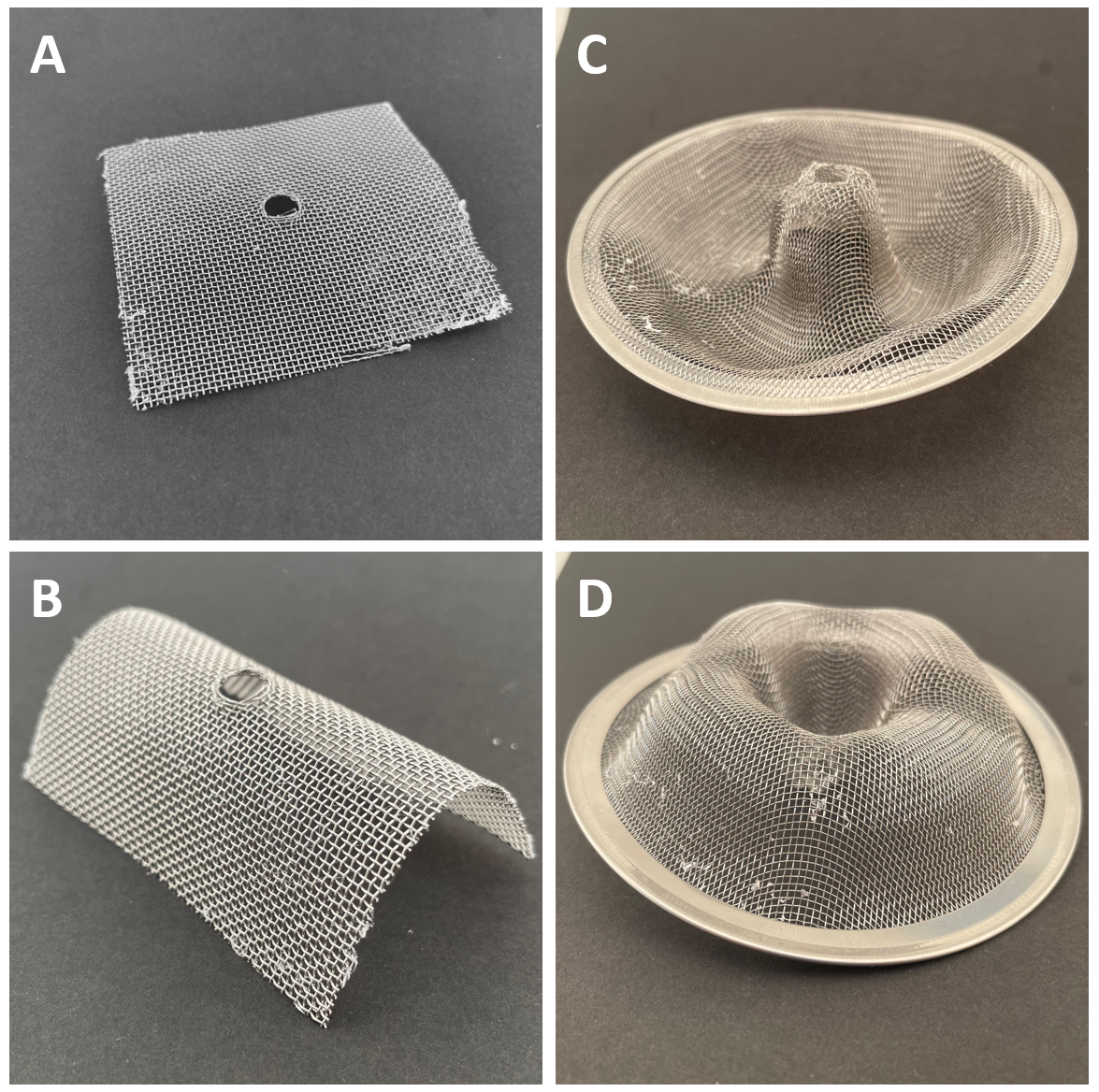

Supplement: Supplementary file 4 [file Image1.TIF]
